# [^18^F]DPA-714 as a biomarker for positron emission tomography imaging of rheumatoid arthritis in an animal model

**DOI:** 10.1186/ar4508

**Published:** 2014-03-13

**Authors:** Géraldine Pottier, Nicholas Bernards, Frédéric Dollé, Raphael Boisgard

**Affiliations:** 1 Inserm, Unité 1023, Université Paris Sud, Orsay 91400, France; 2 Commissariat à l’Energie Atomique et aux Energies Alternatives, Direction des Sciences du Vivant, Institut d’Imagerie Biomédicale, Service Hospitalier Frédéric Joliot, Orsay 91400, France

## Abstract

**Introduction:**

Rheumatoid arthritis (RA) is a chronic disease, affecting 0.5 to 1% of adults in industrialized countries, in which systemic inflammation and synovitis drive joint destruction. [^18^F]DPA-714 is a specific tracer of the 18 kDa translocator protein (TSPO), which is overexpressed on activated macrophages, and proposed as a biomarker of neuroinflammation. Today, diagnosis of patients with early inflammatory arthritis is limited by poor sensitivity and specificity. The present study aims to investigate the potential of [^18^F]DPA-714 to monitor *in vivo* inflammatory processes at a preclinical stage via positron emission tomography (PET).

**Methods:**

RA was induced in Dark Agouti rats by subcutaneous injection of inactivated *Mycobacterium tuberculosis*. Development of arthritis clinical signs was investigated daily and the severity of the disease evaluated. Animals were imaged at the peak of inflammation using [^18^F]DPA-714 and a small-animal PET-CT tomograph.

**Results:**

The first clinical signs appeared at 10 days post-injection, with a peak of inflammation at 20 days. At this time, PET-analyses showed a clear uptake of [^18^F]DPA-714 in swollen ankles, with mean values of 0.52 ± 0.18% injected dose (ID/cc) for treated (*n* = 11) and 0.19 ± 0.09 for non-treated (*n* = 6) rats. A good correlation between [^18^F]DPA-714’s uptake and swelling was also found. Immunohistochemistry showed an enhanced TSPO expression in hind paws, mainly co-localized with the macrophages specific antigen CD68 expressing cells.

**Conclusion:**

These preliminary results demonstrate that the TSPO 18kDa specific radioligand [^18^F]DPA-714 is adapted for the study and follow-up of inflammation linked to RA in our experimental model, suggesting also a strong potential for clinical imaging of peripheral inflammation.

## Introduction

Rheumatoid arthritis (RA) is a chronic inflammatory disorder that can affect many tissues and organs. This disease leads to progressive joint destruction principally due to synovial inflammation, but also to articular complications and functional disability. In addition to causing joint problems, RA can also affect the whole body with fevers and fatigue. RA is a systemic autoimmune disease that also leads to many disorders in organ systems and is associated with other diseases, including infections, malignancies and cardiovascular diseases. One percent of the world's population today is affected by RA with a repartition much more common in women than in men with ages ranging from 40 to 60 years [1]. Smoking is the main environmental risk factor for developing RA. Other potential risks, including alcohol abuse, vitamin D status, contraception use and coffee intake, have been reported too [2].

The detection of RA is important in clinical strategies that include early treatments which may slow down the disease progression and improve the long-term clinical outcome. Criteria proposed in 1987 by the American College of Rheumatology (ACR) 1987 are rather limited by the poor sensitivity and specificity for the classification of patients with early RA [3]. These criteria include morning stiffness, arthritis of three or more areas, arthritis of hand joints (>1 swollen joint), rheumatoid nodules, serum rheumatoid factor, radiographic changes (erosions). However, early events failed to be detected by those means. Until today, identification of bone changes in RA is traditionally the domain of plain radiography. In parallel to this, other techniques such as Magnetic Resonance Imaging and Ultrasound have demonstrated a superior sensitivity for the detection of erosive changes around the bone. Several teams have shown that subclinical arthritis can be detected by advanced imaging techniques such as sonography or power Doppler [4-6]. The development of non-invasive, highly sensitive and 3D-imaging-based methods for detecting early arthritis could also offer an opportunity to initiate treatments at very early stages to the patient.

Molecular- and nuclear-based imaging techniques offer a large scale opportunity for detection of diseases. These techniques are based on the use of tracers labeled with radioactive isotopes and allow non-invasive *in vivo* detection of different physiologic and pathologic phenomena with high sensitivity. Positron Emission Tomography (PET) is one of those that are increasingly used to diagnose and characterize disease activity in the setting of inflammatory disorders such as RA [7-10]. PET is even more sensitive than Single Photon Emission Computed Tomography (SPECT) and can also provide quantitative measurements. The low spatial resolution can make assigning the signal to specific anatomical structures difficult and can be partially compensated by combining PET with CT (X-ray Computed Tomography). [^18^F]Fluorodeoxyglucose ([^18^F]FDG), a radiofluorinated analogue of glucose and the most widely used radiopharmaceutical worldwide today, was proposed for imaging RA [11,12]. Using this radiotracer, inflamed joints could be detected even though [^18^F]FDG is not a specific marker of inflammation. Macrophage infiltration has been identified in early stages of RA [13], and therefore a specific tracer of such a process would be more specific and possibly also enable an earlier detection of inflammation. Recently, expression of the folate receptor has been investigated in a rat model of RA using [^18^F]fluoro-PEG-folate [14] illustrating the interest for molecular imaging in this type of pathology.

The 18kDa translocator protein (TSPO), previously known as the peripheral benzodiazepine receptor (PBR), is located in the outer mitochondrial membrane [15]. This protein is up-regulated in inflammatory processes, making radiolabeled TSPO ligands attractive imaging probes in a multitude of pathologies. Different TSPO ligands have already been used as imaging probes in different animal models of human diseases such as cancer [16], cerebral ischemia [17], lung [18] and liver diseases [19].

Today, [^11^C]PK11195 is still considered as the reference radioligand for imaging *in vivo* TSPO expression mainly in central nervous system disorders and rarely in the case of peripheral inflammatory diseases. Numerous publications in the recent literature have, however, highlighted the limitations of this radiotracer for quantitative *in vivo* measurement of TSPO expression [20] and several novel TSPO radioligands have been developed today as alternatives to the use of [^11^C]PK11195 [21-23]. Among them, the pyrazolo [1,5] pyrimidine acetamide DPA-714, labeled with fluorine-18 ([^18^F]DPA-714) is one of the most promising compounds. For the moment, [^18^F]DPA-714 is mainly used to image brain injury in various animal models [17,24-26] but also in the field of oncology with applications in breast cancer [27] and glioma [28]. Interest for the molecular imaging of peripheral inflammation has also been investigated recently in non-alcoholic fatty liver disease in rats [19] and lungs by the same team [18] using another radiolabelled TSPO ligand ([^18^F]FEDAC). Recently, [^11^C]PK11195 has been shown to be of potential interest in imaging arthritis in humans [7,10,29]. The visualization of macrophages using [^11^C]PK11195 PET seems to be useful for detecting early synovitis and for monitoring disease evolution during treatment.

The present study aims to investigate the potential of [^18^F]DPA-714 PET to image and quantify *in vivo* peripheral inflammation in an autoimmune adjuvant-induced RA rat model.

## Methods

### Animals

Male Dark Agouti rats (216.7 g ± 16.4 g) were purchased from Centre d’Elevage René Janvier (Saint Berthevin, France), housed and acclimatized for one week before treatment with free access to food and water. Animal studies were conducted in accordance with the French legislation and European directives (2010/63/UE) on the uses of animals in research. Experiments were done in the approved French laboratory D 91 471 105 from 2 August 2012, under the supervision of the institutional ethical committee (CETEA DSV, recorded in 6 June 2011 under No. 44 by CNREEA, National Committee for ethical reflection on animal experimentation).

### Preparation of CFA (complete Freund’s adjuvant)

*Mycobacterium tuberculosis* H37 Ra (Mtb) and incomplete Freund’s adjuvant (IFA) were purchased from Difco Laboratories (USA). CFA was prepared as follows: IFA (20 mL) was drop-wise added, with continuous mixing, to finely crushed Mtb (100 mg). The resulting oily preparation (Mtb: 5 mg/mL) may be temporarily stored at -20°C if not readily used.

### Induction of RA

Rats were anesthetized with an isoflurane/oxygen mixture (2 to 4%). Induction of RA was performed for each rat by intra-dermal injection at the base of the tail of 100 μL (single dose) of the above reported CFA.

### Clinical evaluation of RA

Rats were observed daily. Animals were weighed and development of arthritis clinical signs investigated every two days. The clinical severity of RA was evaluated according to the following scale: 1 = detectable swelling in one joint; 2 = swelling in two joints; 3 = swelling in three joints; 4 = severe swelling of the entire paw. The maximum score per animal for the four paws was 16. Each observation was done under short anesthesia using an isoflurane/oxygen mixture (2 to 3%).

### [^18^F]DPA-714 preparation

DPA-714 was labeled with fluorine-18 (half-life, 109.8 minutes) at its 2-fluoroethylmoiety using a tosyloxy-for-fluorine nucleophilic aliphatic substitution, according to slight modifications of procedures already reported [30,31], and using a commercially available GE TRACERLab FX-FN synthesizer [32]. Ready-to-inject, >99% radiochemically pure [^18^F]DPA-714 (formulated in physiological saline containing less than 10% of ethanol) was obtained with 15 to 20% non-decay-corrected yields and specific radioactivities at the end of the radiosynthesis ranging from 37 to 111 GBq/μmol. Injected doses (MBq and nmoles, mean ± SD) will be given within the text.

### PET/CT imaging and data analysis

PET/CT scans were performed using small-animal INVEON (Siemens, Munich, Germany) tomography. This system combines both PET and CT modalities under the control of a unique workstation, including CT-based attenuation correction which allows for superior quality PET images. PET/CT scans were performed around the peak of the disease (20 days) following CFA-injection in all animals showing a clinical score ranging from 1 to 16 as well as in control animals (free of injection).

Animals were anesthetized with an isoflurane/oxygen mixture (2 to 4%) and maintained normothermically using a heating pad. Injections of [^18^F]DPA-714 were made in the caudal lateral vein using a 24-gauge catheter.

Dynamic acquisitions were done as follows: imaging started at the time of injection of [^18^F]DPA-714 (38.0 +/ -3.1 MBq/rat corresponding to 2.2 +/ -0.8 nmol by injection) and continued for 60 minutes (n = 5). Static acquisitions were obtained 45 minutes after injection of the radiotracer and lasted for a period of 15 minutes (n = 6). In displacement studies (n = 3) animals received, 30 minutes post [^18^F]DPA-714-injection, a solution of DPA-714 (5 mg/kg) intravenously. Data acquisition was continued for another 30 minutes.

All PET-measurements were performed with a time coincidence window of 3.432 ns and the energy levels of discrimination at 350 keV and 650 keV. The list mode data files were histogrammed into 3D-sinograms with a maximum ring difference of 79 and a span of 3. Images were reconstructed using ASIPro (Siemens) with the FORE + OSEM2D algorithm (16 subsets and 4 iterations).

Image analysis and quantification of radioactivity in volumes of interest were performed using Brain-Visa/Anatomist version 3.1 [33]. Regions of interest (ROIs) were drawn on the ankle joints. These ROIs were then projected onto all dynamic frames, thereby creating time activity curves for each ROI. Radioactivity uptake values were quantified in Bq per cubic cm (cc) of tissue, corrected for fluorine-18-decay and converted into the percentage of injected dose per cubic cm (% ID/cc).

### Histology

After rat euthanasia (lethal dose of pentobarbital 150 mg/kg), the paws were then collected and fixed in 10% (v/v) buffered formalin phosphate (Labonord, France) for 24 h to 48 h, before being decalcified using Immunocal (Decal Chemical Corporation, USA) for two weeks. After decalcification, the paws were frozen in liquid nitrogen and stored at -80°C before being sliced with a cryotome (Leica CM3050, Germany). Finally, the ankle joints were cut in a sagittal plane and sectioned at 5 μm.

Selected sections were stained with Mayer’s hematoxylin solution (Sigma, USA) and eosin-y (Labonord, France) or directly used for immunohistochemistry.

### Immunohistochemistry

Immunohistochemistry (IHC) was performed on 5 μm frozen sections of paw slices. Slices were placed for 15 minutes in fixative containing 4% paraformaldehyde in PBS (phosphate-buffered saline), then removed and placed in 50 mM aq. NH_4_Cl for 5 minutes. Slices were then placed in PBS containing 5% BSA (bovine serum albumin) (Merck, Germany) and 0.5% Tween 20 (Sigma, USA) for five minutes at room temperature (RT). Finally, slices were incubated (1 h, RT) with the following primary antibodies in solution in the above mentioned buffer: mouse anti-CD68 (MRA341R, 1:100; Serotec, Germany), goat anti-Iba 1 (ab5076, 1:100; Abcam, UK) and rabbit anti-TSPO (NBP1-95674, 1:1,000; Novus Biologicals, UK).

Slices were then washed three times with PBS and then incubated (30 minutes, RT) with Alexa Fluor-546 donkey anti-mouse (A10036, 1:1,000; Invitrogen, France), Alexa Fluor-647 donkey anti goat (A21447, 1:1,000; Invitrogen, France), Alexa Fluor-488 goat anti-rabbit (A11034; 1:1,000; Invitrogen, France), respectively, in a solution of PBS containing 5% BSA and 0.5% Tween 20.

Slices were again washed three times in PBS and then mounted with a DAPI Kit (P36931 Invitrogen, France).

The number of CD68-, TSPO- and Iba1-positive cells were counted under the ×20-objective of an Axio observer Z1 microscope (Zeiss, France) on seven fields of view taken from three adjacent sections per rat.

### Statistical analysis

Statistical analyses were conducted using Microsoft Excel software. A significant difference between arthritic and control groups was determined at each point by the one-tailed Student’s *t*-test. A *P*-value of less than 0.05 was deemed as significant.

## Results

### Rheumatoid arthritis model

**Figure 1:**
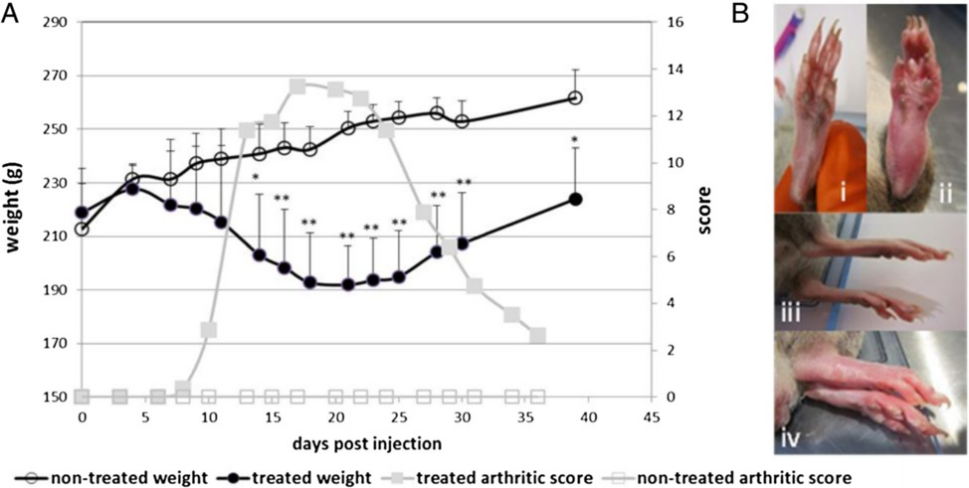
**Model of arthritis in Dark Agouti rats. (A)** Mean arthritic score and weight evolution at different times after injection of *Mycobacterium tuberculosis* H37 (Mtb, at the base of the tale) in non-treated (n = 6) and treated (n = 11) Dark Agouti (DA) rats. **(B)** Illustrations of paws of non-treated (healthy) DA rats (i, iii) and treated (*Mycobacterium tuberculosis* H37 Ra (Mtb)) rats (ii, iv) at 14 days. *P <0.05; **P <0.001.

An arthritic model was induced by a single dose injection of 0.5 mg of *Mycobacterium tuberculosis* H37 Ra (Mtb) in male Dark Agouti rats (DA rats). During the disease progression, two parameters were followed: the bodyweight and the joint swelling. These parameters are both reported as systemic RA symptoms. Figure 1A illustrates the typical clinical course of the RA in DA male rats after injection of Mtb. The initial signs of arthritis appeared around 10 to 13 days after the injection, mostly as swelling of the metatarsophalangeal or ankle joints of the hind paws. The inflammation progressed to the entire hind paw (Figure 1B) and at a later stage, the joints in the front paws also became inflamed. The inflammation’s peak appeared at around 20 days after Mtb-injection; thereafter, the swelling progressively declined and disappeared by about 40 days after Mtb-injection. The body weight of treated animals started to decrease six to seven days post-Mtb-injection. A loss of weight of up to 18.5% could be observed. This loss of weight stopped with the decrease of the inflammation. No apparent clinical symptoms occurred for the non-treated animals. As expected for this group, a regular weight gain was observed.

### Histological evaluation of the RA model

**Figure 2:**
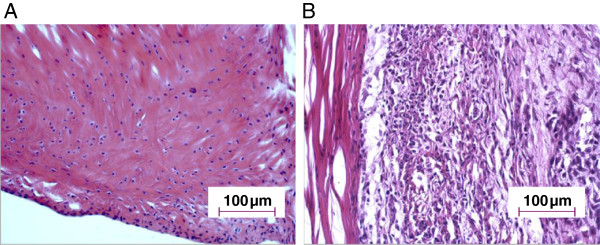
**Histological characterization of the RA model in DA rats.** Tissue slides from joints of non-treated rats **(A)** and joints from Mtb-treated rats **(B)** were analyzed at 20 days post-injection using Hematoxylin and Eosin stained sections from ankle joints (hind paw). The histological architecture shows synovial hyperplasia and infiltration of inflammatory cells. (Magnification x200).

**Figure 3:**
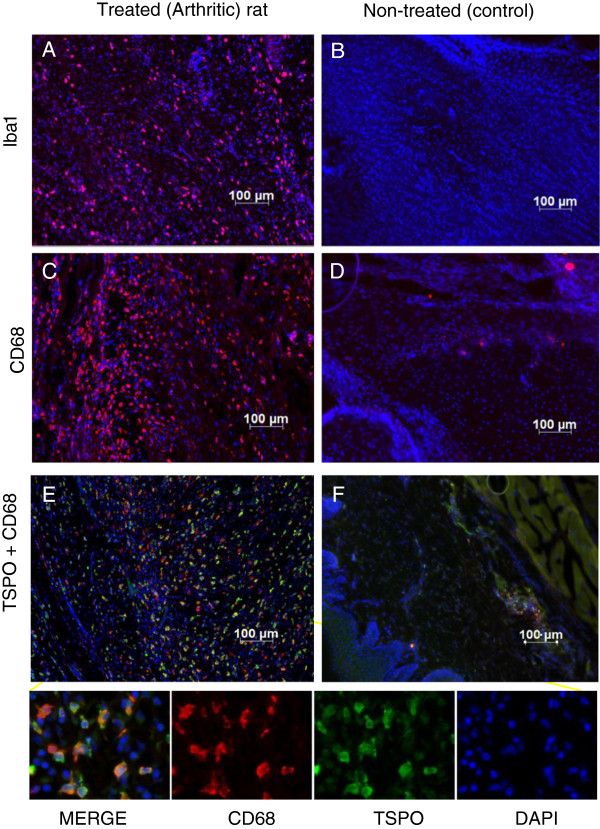
**TSPO expression in hind paws of RA rats is associated with activated macrophages.** Slide tissues of treated (RA) **(A and C)** and non-treated (control) rats **(B and D)** were stained with CD68 (red) or Iba1 (red) for activated macrophages. Nucleuses were colored with DAPI (blue). Representative IHC staining of TSPO positives cells (green) from treated (RA) rats **(E)** and non-treated (control) rats **(F)** and the colocalization with CD68 cells (red) are also shown. IHC, immunohistochemistry; RA, rheumatoid arthritis; TSPO, translocator protein.

The arthritic effects of the RA model were analyzed on histological joint sections stained with Hematoxylin and Eosin at 20 days post-Mtb-injection. Severe signs of inflammation could be detected in treated rats (Figure 2B) with an increased amount of infiltrated inflammatory cells (such as lymphocytes and macrophages) while only scarce proliferation was observed in non-treated rats (Figure 2A). Immunohistochemical staining on joint sections was also performed in order to characterize TSPO expressing cells. Specific antibodies directed against CD68 and Iba 1 confirmed the significant increase of activated macrophages in treated (RA) rats (Figure 3A, C) compared to non-treated rats (Figure 3B, D). TSPO-labeling on the RA rats’ joints slides (Figure 3E) was mainly co-localized with CD68-labeling. Cell counting showed that 23% of the cells present in the ankle were CD68 positive. Among them, 95% expressed TSPO and only about 5% were TSPO negative. In the inflamed ankle, around 4% of the cells were visualized expressing only the TSPO (CD68-). In non-treated animals, these cell activations were not visible in joint slides and the proportion of CD68 positive cell was less than 1% (Figure 3F).

### Uptake of [^18^F]DPA-714 in paws (ankles) of RA rats and non-treated rats

**Figure 4:**
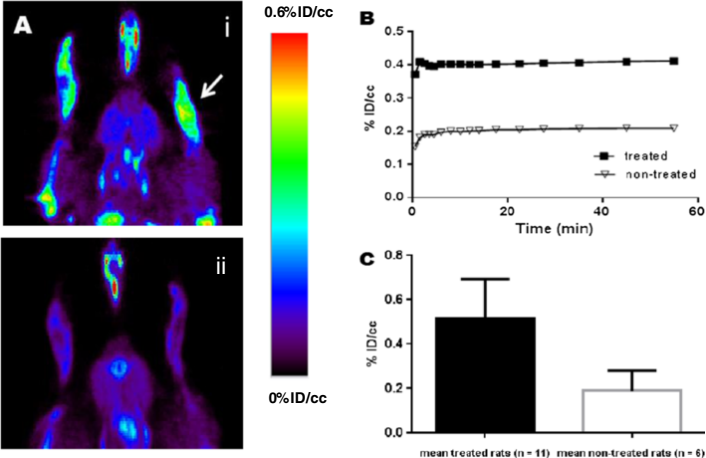
**Enhanced uptake of [**^**18**^**F]DPA-714 in paws (ankle) of RA rats versus control using microPET. (A)** Representative images in coronal sections of the increased uptake of [^18^F]DPA-714 in the ankles of a Mtb-treated (RA) rat (**A**, i) compared to non-treated (control) rat (**A**, ii) at 20 days. **(B)** Kinetics of [^18^F]DPA-714 uptake for non-treated and Mtb-treated (RA) rats. **(C)** Mean of [^18^F]DPA-714 uptake at 55 minutes for treated (RA) (n = 11) versus non-treated (control) (n = 6) group. **P = 0.00008. Mtb, *Mycobacterium tuberculosis* H37 Ra; PET, positron emission tomography; RA, rheumatoid arthritis.

The capacity to detect and quantify peripheral inflammation lesions in the hind paw of rats *in vivo* was evaluated by microPET imaging using [^18^F]DPA-714. Figure 4A shows an image of a coronal section in which increased uptake of [^18^F]DPA-714 in ankles of Mtb-treated (RA) rat (4A, i) compared to non-treated (control) rat (4A, ii) at 20 days. Both images are represented using the same color scale. A sharp increase of the radiotracer’s uptake in the ankles of the treated rat is clearly illustrated (indicated by white arrows). A typical time-activity curve observed in the hind paw of a treated rat at 20 days post-Mtb-induction, scored 4 (clinical severity grade) is represented in Figure 4B. As shown, the uptake of [^18^F]DPA-714 rapidly reached a maximum and steady value, only a few minutes following the i.v. injection of the radiotracer and remained stable throughout the 60-minute acquisition. The mean [^18^F]DPA-714 uptake value in treated animals was more than twice that of the non-treated animals. Values reaching 0.52 ± 0.18 and 0.19 ± 0.09% ID/cc were found for treated animals (n = 11 with a score ranging from 5 to 16) and non-treated animals (n = 6), respectively (Figure 4C). These results were statistically significant with a *P*-value of 0.00008 (t-test).

### *In vivo* specificity

**Figure 5:**
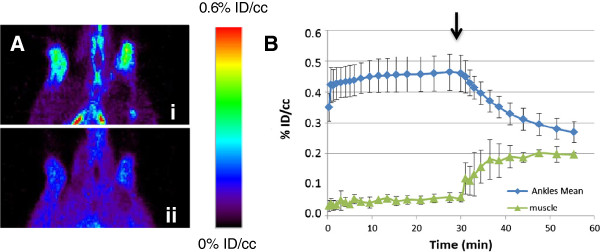
**Displacement experiment by unlabeled DPA-714. (A)** Visualization of the same animal (treated) just before injection of unlabeled DPA-714 (**A**, i) and 15 minutes after injection of unlabeled DPA-714 (**A**, ii). **(B)** Kinetics of [^18^F] DPA-714 uptake in the ankles and muscle, followed by DPA-714. Arrow indicates time of displacement, 30 minutes after radiotracer injection.

To eliminate the possibility that [^18^F]DPA-714 uptake was driven by unspecific processes, and also to evaluate at the same time the specificity of this binding, animals were injected 30 minutes post-[^18^F]DPA-714 administration, with a large excess (5 mg/kg of body weight) of DPA-714 (unlabeled). Figure 5A permits us to compare the same animal just before injection (5A, i) and 15 minutes after injection of unlabeled DPA-714 (5A, ii). An important decrease of the signal in the ankles as well as an increase of the background signal (illustrated by the higher signal in the muscle) could be noticed following the intravenous injection of non-labeled DPA-714. This increase of background could mainly be explained by the release of [^18^F]DPA-714 from the heart, lungs and spleen, three organs characterized by a high level of TSPO expression. Figure 5 illustrates the mean curve of displacement carried out on four different animals with similar grades of the disease. The mean radioactivity in this group of animals is around 0.46 +/- 0.06% of ID/cc just before the displacement challenge and decreased to 0.27 +/- 0.03% of ID/cc at 30 minutes after the injection of unlabeled DPA-714. This variation corresponds to a displacement around 45% of the initial signal present in the ankles. During the same time, the mean value in the muscle increased from 0.06 +/-0.01% ID/cc to 0.20 +/- 0.01% ID/cc corresponding to a variation around 300%.

### Correlation between the uptake of [^18^F]DPA-714 and the severity of the disease

**Figure 6:**
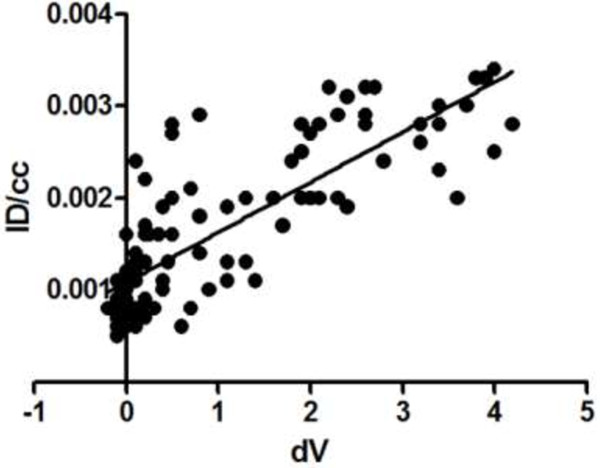
**Correlation between uptake and severity.** For each animal, the ID/cc and respective volume of the ankles were plotted. The linear regression, using the least square method, gives a determination coefficient R^2^ equal to 0.65.

To analyze the relation between the uptake of the radiotracer and the inflammatory status, the ID/cc and the volume of the ankle were plotted as presented in Figure 6. A linear regression, using the least square method, gave a determination coefficient of R^2^ equal to 0.65 illustrating a good relation between the uptake of this radiotracer and the severity (swelling) of the disease.

## Discussion

The present study used a readily accessible, small molecular weight, fluorine-18-labeled compound, coded DPA-714, as the radiotracer for the inflammatory processes. This TSPO radioligand has been largely used to monitor neuroinflammation. The results published recently in various articles illustrate the potential of [^18^F]DPA-714 to provide quantitative information about the expression of TSPO with correlation between the radioactive signal obtained by PET imaging and the molecular expression of the target, at a cellular level, obtained by Western blot analysis [16,24]. In our inflammatory model, biochemical analysis could not be easily performed due to the presence of bones and cartilages. For this reason we chose to illustrate the increase of TSPO using IHC. This approach, done on fixed tissues, required a decalcification step to permit the slicing of the articular tissues. These analyses were done on control, mild and severe inflammatory grade permitting us to visualize an increasing number of CD68 and TSPO positive cells (data not shown). The combination of both [^18^F]DPA-714 PET imaging and IHC clearly illustrated the increase of TSPO expression in the ankles of treated rats in comparison to non-treated animals. IHC using CD68 showed the important infiltration of white blood cells, mainly macrophages, in the ankle. The CD68 positive cells represented around 25% of the cells counted on tissue samples from inflamed ankles. Co-labeling using a TSPO antibody showed that 95% of these CD68 marked cells were also TSPO positive. TSPO is expressed in mitochondria whereas CD68 is mainly localized in cytoplasmic granules. This non-overlap, adding to the counting error incertitude, does not permit us to affirm that some CD68 positives cells are really TSPO negatives. These types of cells (TSPO-; CD68+) represent less than one percent of total cells counted in the different fields of view for this quantitative approach. On the other hand, around 4% of total cells by field of view are TSPO + and CD68-. This cell population could represent other immune cells, such as lymphocytes or macrophages as suggested by the presence of Iba1+/CD68- or MRP8+/CD68- cells.

Conventional radiography is nowadays considered as the gold standard in RA imaging for the evaluation of structural damages. The main limitation with this technique is that its sensitivity is considered to be low and also that it is not able to assess the disease activity.

Recently, an international group of expert rheumatologists, radiologists and methodologists published some recommendations for the use of imaging of the joints in the clinical management of rheumatoid arthritis [34]. They compared the potential of different imaging techniques for diagnosis of RA, detecting inflammation and damage, predicting outcome and response to treatment, monitoring disease activity, progression and remission. These techniques, conventional radiography, MRI, CT, dual emission X-ray absorptiometry, digital X-ray radiogrammetry are anatomical images based mainly on density alteration of bones and cartilages. Two other techniques were included in this comparative study, SPECT and PET imaging. These two nuclear-based approaches mainly provide information on biological functions (molecular imaging) as opposed to anatomical imaging. In arthritis, anatomical alterations of bone, cartilage or at least severe stage tendon swelling, represents a late stage of the disease with clinical symptoms illustrative of a long molecular process of inflammation. Concerning the diagnosis of RA, precocity has been described as an important point to implement appropriate therapeutics and thus limit structural alterations and delayed invalidity associated with this disease. Molecular imaging, such as TSPO PET imaging, represents a possibility of following early events in the physiopathology of RA. It has, therefore, been proposed that [^11^C]PK11195 PET imaging could be used for detecting early synovitis and for monitoring the evolution of the disease during treatment [7,10]. The present work, performed in a rat RA model, demonstrates that [^18^F]DPA-714 can be used to image inflammation of joints non-invasively. The specificity of the uptake was assessed here by intravenous injection of a large amount of unlabeled DPA-714. This injection displaced specific binding of [^18^F]DPA-714 by saturation of TSPO binding site by a non-labelled compound. The phenomena took place in an inflamed area but also in every organ with a physiological high content of TSPO inducing plasma release of a large quantity of [^18^F]DPA-714. This effect is illustrated by the huge increase in an unspecific signal quantified in the muscle with a variation from 0.06 to 0.20% of ID/cc (Figure 5B). In the inflamed ankles, the displacement leads a decrease of the signal around 45%. After administration of unlabeled DPA-714, the ankle-to-muscle ratio in these animals was around 1:4 while this ratio was 7:8 before administration. This value illustrates that the signal quantified in the ankle after displacement is largely due to background corresponding to free [^18^F]DPA-714 (not bound to the TSPO 18kDa). In the inflamed areas it has been shown that the vascular density is also increased. This high vascular density can increase the volume of blood in the considered area and participate in the higher signal in comparison to normal ankles or muscle use as the reference area. Local inflammation also induces edema. Edematous areas are well known to present high oncotic pressure inducing extravasation of macromolecules. This phenomenon was not described for small ligands, such as DPA-714, with a molecular weight around 400 g/mol. This hypothesis is certainly not the explanation concerning the remaining radioactivity present in the ankle after displacement. The level of radiotracer accumulation in the swollen hind paws correlates to the thickness of the ankles, and is chosen as macroscopic criteria for the severity of the disease (Figure 6). In this graph, uptake results were expressed as density by number of the receptors by volume unit here extrapolated as Bequerel/cc corrected by the ID/cc. The fact that the increase of ankle volume was linked to an increase of the TSPO density (for example, [^18^F]DPA-714’s uptake) underlines the relation between the severity of the disease and the level of TSPO expression. This functional information represents an important point concerning the ability of imaging techniques to evaluate disease activity, a parameter simply not accessible using conventional anatomical imaging approaches. Another criterion that represents a large interest for rheumatologists is the potential of such non-invasive imaging tools and techniques to predict the evolution of the disease.

## Conclusion

The results of the present work demonstrates that the use of the TSPO-targeting PET-ligand [^18^F]DPA-714 is well-adapted to the study of peripheral inflammation in an experimental rodent model of RA. [^18^F]DPA-714 PET imaging may provide new objective parameters to evaluate the early stages of the disease and to determine disease activity, also suggesting a strong potential of this technique for clinical investigation of peripheral inflammation.

## Abbreviations

[18F]FDG: [^18^F]Fluorodeoxyglucose; ACR: American College of Rheumatology; BSA: Bovine serum albumin; CFA: Complte Freund's adjuvant; CT: X-ray computed tomography; ID/cc: Injected dose; IFA: Incomplete Freund’s adjuvant; IHC: Immunohistochemistry; Mtb: *Mycobacterium tuberculosis* H37 Ra; PBS: Phosphate-buffered saline; PET: Positron emission tomography; RA: Rheumatoid arthritis; ROIs: Regions of interest; RT: Room temperature; SPECT: Single photon emission computed tomography; TSPO: Translocator protein.

## Competing interests

The authors declare that they have no competing interests.

## Authors’ contributions

GP helped to design the study, performed the experiments, analyzed and interpreted data and drafted the manuscript. NB participated in imaging acquisitions and critically reviewed the manuscript. FD participated in [^18^F]DPA-714 syntheses and was involved in revising the manuscript. RB designed the study, participated in data analysis and drafted the manuscript. All authors read and approved the final version of the manuscript.
